# Identifying the Evolutionary Building Blocks of the Cardiac Conduction System

**DOI:** 10.1371/journal.pone.0044231

**Published:** 2012-09-11

**Authors:** Bjarke Jensen, Bastiaan J. D. Boukens, Alex V. Postma, Quinn D. Gunst, Maurice J. B. van den Hoff, Antoon F. M. Moorman, Tobias Wang, Vincent M. Christoffels

**Affiliations:** 1 Department of Anatomy, Embryology & Physiology, Academic Medical Center, University of Amsterdam, Amsterdam, The Netherlands; 2 Department of Biological Sciences, Zoophysiology, Aarhus University, Aarhus, Denmark; Leiden University Medical Center, The Netherlands

## Abstract

The endothermic state of mammals and birds requires high heart rates to accommodate the high rates of oxygen consumption. These high heart rates are driven by very similar conduction systems consisting of an atrioventricular node that slows the electrical impulse and a His-Purkinje system that efficiently activates the ventricular chambers. While ectothermic vertebrates have similar contraction patterns, they do not possess anatomical evidence for a conduction system. This lack amongst extant ectotherms is surprising because mammals and birds evolved independently from reptile-like ancestors. Using conserved genetic markers, we found that the conduction system design of lizard (*Anolis carolinensis* and *A. sagrei*), frog (*Xenopus laevis*) and zebrafish (*Danio rerio*) adults is strikingly similar to that of embryos of mammals (mouse *Mus musculus*, and man) and chicken (*Gallus gallus*). Thus, in ectothermic adults, the slow conducting atrioventricular canal muscle is present, no fibrous insulating plane is formed, and the spongy ventricle serves the dual purpose of conduction and contraction. Optical mapping showed base-to-apex activation of the ventricles of the ectothermic animals, similar to the activation pattern of mammalian and avian embryonic ventricles and to the His-Purkinje systems of the formed hearts. Mammalian and avian ventricles uniquely develop thick compact walls and septum and, hence, form a discrete ventricular conduction system from the embryonic spongy ventricle. Our study uncovers the evolutionary building plan of heart and indicates that the building blocks of the conduction system of adult ectothermic vertebrates and embryos of endotherms are similar.

## Introduction

**Figure 1:**
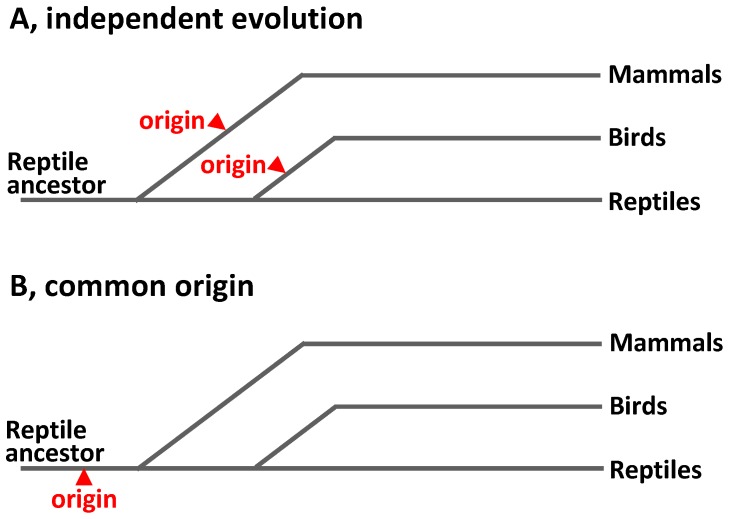
Background and hypothesis. (A) Anatomical works concluded that the specialized cardiac conduction system evolved independently in mammals and birds because similar structures could not be found in ectothermic vertebrates. (B) We are testing the hypothesis that a primordial version of the specialized cardiac conduction system can be found in ectothermic vertebrates.

The hearts of mammals and birds maintain high rates of contraction [1] that in concert with high systemic blood pressures accommodate their high rates of oxygen consumption due to their endothermic state [2]. The high heart rates, the timing of sequential atrial and ventricular contractions and the rapid spread of the activating impulse over the avian and mammalian ventricles are possible because of a specialized cardiac conduction system [3]. However, while the sequential activation of the cardiac chambers and appropriate matching of the atrial and ventricular contractions are similar across all vertebrate groups, there is no anatomical evidence for a specialized conduction system in hearts of reptiles or other ectothermic vertebrates [4], [5]. Because mammals and birds evolved independently from reptilian ancestors, the evolutionary origin of their specialized conduction systems has remained unclear; either their conduction systems evolved independently or primordial components of the system were already present in the ancestral reptiles (Fig. 1).

Like the hearts of ectothermic vertebrates, embryonic mammalian and avian hearts also exhibit regulated sequential activation patterns in the absence of a morphological conduction system [2], [6], [7]. This suggests that the functional components for conduction system are established early in development and in evolution, but are not represented by anatomically distinguishable components as in the mature hearts of endothermic vertebrates. Instead, the components may reflect an intrinsic part of the building plan of the heart.

**Figure 2:**
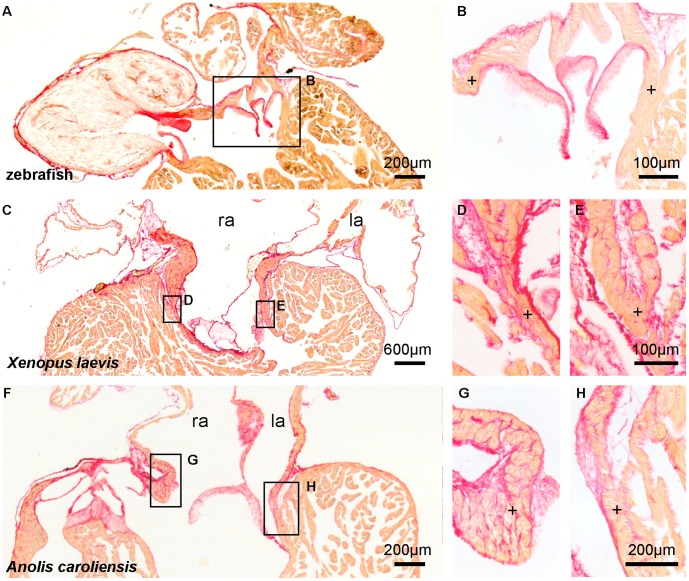
The atrioventricular junction in ectotherms is not interrupted by an insulating plane. Picro-sirius red stain for collagen (red) on 10 µm sections of hearts of adult ectotherms showing the atrioventricular canal to be in full communication (+) with the ventricle. l(r)a, left(right) atrium.

**Figure 3:**
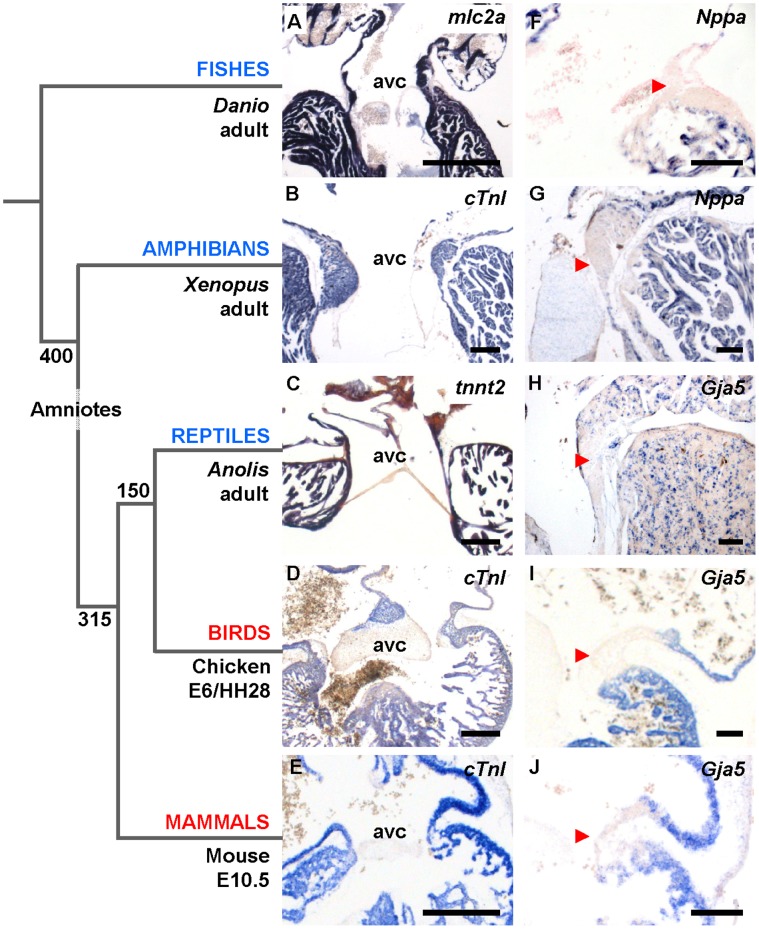
The phenotype of the slow propagating atrioventricular canal is evolutionary conserved. Numbers in the phylogenetic tree indicate time in millions of years since major splits in tetrapod evolution. (A–E) The hearts of mature ectotherms (blue) and embryonic endotherms (red) maintain complete muscular connection in the atrioventricular canal (avc). (F–J) Markers of fast propagating chamber myocardium (*Nppa* and *Gja5*) are absent from the atrioventricular canal (arrowheads). Note that the specimen in H is contracted, obscuring the spongy design otherwise visible. Scale bars in (A–E), 300 µm; (F–J), 100 µm.

*Tbx2* and *Tbx3* belong to an ancient family of transcription factors [8] expressed in the embryonic atrioventricular canal from human to primitive fish [3], [9]. In the embryo, the atrioventricular canal delays the impulse from atrium to ventricle. *Tbx2/3* suppresses chamber genes including *Nppa* and *Gja5*, encodes for connexin40 that is required for fast conduction [10], and hence inhibits differentiation of the atrioventricular canal to fast-conducting chamber myocardium [3], [11]. *Tbx3* remains expressed in the mature conduction system components of mammals, including the atrioventricular node that derives from the atrioventricular canal [12]. *Bmp2/4* are expressed in the atrioventricular canal of early embryonic mammals, birds and fish, and are crucial for activation of *Tbx2/3* (Fig. S1) [13]–[17]. Hence, *Tbx2/3*, *Bmp2/4* and *Gja5/Nppa* represent evolutionary conserved positive and negative markers, respectively, that discriminate the embryonic slow-conducting atrioventricular myocardium and fast-conducting chamber myocardium. The ventricular His-Purkinje system of mammals and birds is specifically marked by expression of *Gja5* (and *Nppa* in mammals) [3], [10].

Here, we carried out *in situ* hybridization analysis using evolutionary conserved genetic markers and provide a three-dimensional reconstruction of the key components of the conduction system. The ventricular conduction pattern was visualized using optical imaging of activation. The cardiac expression and conduction patterns of a reptile were then compared to those of mammals, chicken, and other ectothermic vertebrates, frog and fish. We find an anatomic, genetic and physiologic conserved building plan where hearts of adult ectothermic vertebrates are similar to embryos of the endothermic mammals and birds. This indicates that primordial components of the cardiac conduction system were present in the ancestral reptiles.

## Materials and Methods

All experimental procedures on adult material complied with national and institutional guidelines and were approved by Institutional Animal Care and Use Committee of the University of Amsterdam. The approval is registered as “DAE101617” for optical mapping of the ectotherms and “DAE101532” for optical mapping of developing mice. In The Netherlands experiments with non-mammalian embryos (that are not autonomously viable) do not require approval from the Institutional Animal Care and Use Committee.

**Figure 4:**
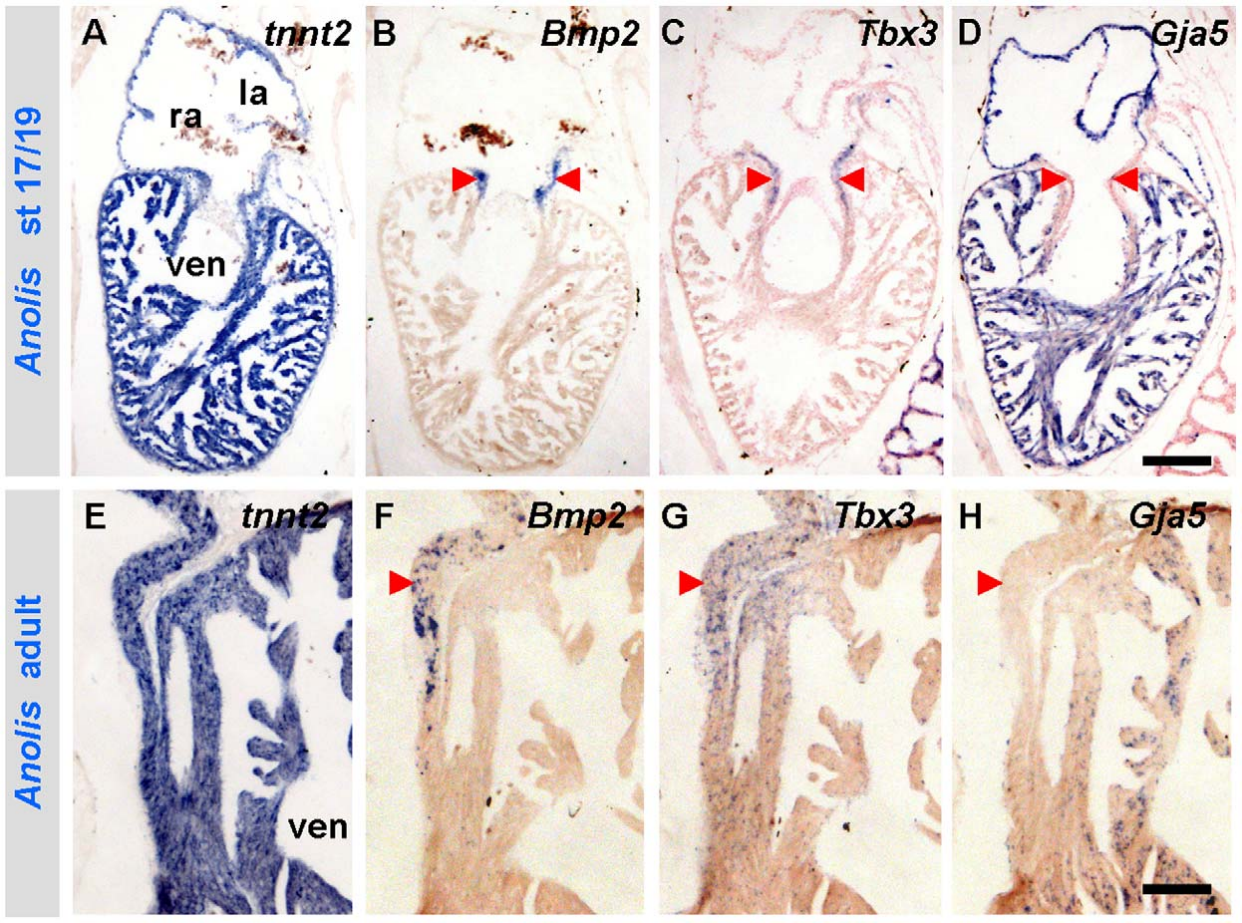
The developmental gene programme of amniotes is maintained in the mature heart of *Anolis*. (A–D) Stage 17/19 *Anolis* hearts show complementary expressions of *Bmp2* and *Tbx3* to *Gja5* in the developing myocardial atrioventricular canal (arrowheads). (E–H) The developmental expression of *Bmp2*, *Tbx3* and *Gja5* is maintained in the mature myocardial atrioventricular canal (left side shown). la, left atrium; ra, right atrium; ven, ventricle. Scale bars are 100 µm.

### Animals

Adult zebrafish were provided by the Hubrecht Laboratory, Utrecht, the Netherlands, and adult *Xenopus laevis* from Leiden University, the Netherlands. Mice and *Xenopus laevis* embryos were raised in the AMC. Green and brown anole (*Anolis carolinensis* and *A. sagrei*) eggs and adults and fertilized chicken eggs, were bought commercially in the Netherlands. *Xenopus* embryos were staged according to Nieuwkoop [18], *Anolis* embryos according to Sanger et al. [19], chicken according to Hamburger and Hamilton [20] and mice from days post coitus.

**Figure 5:**
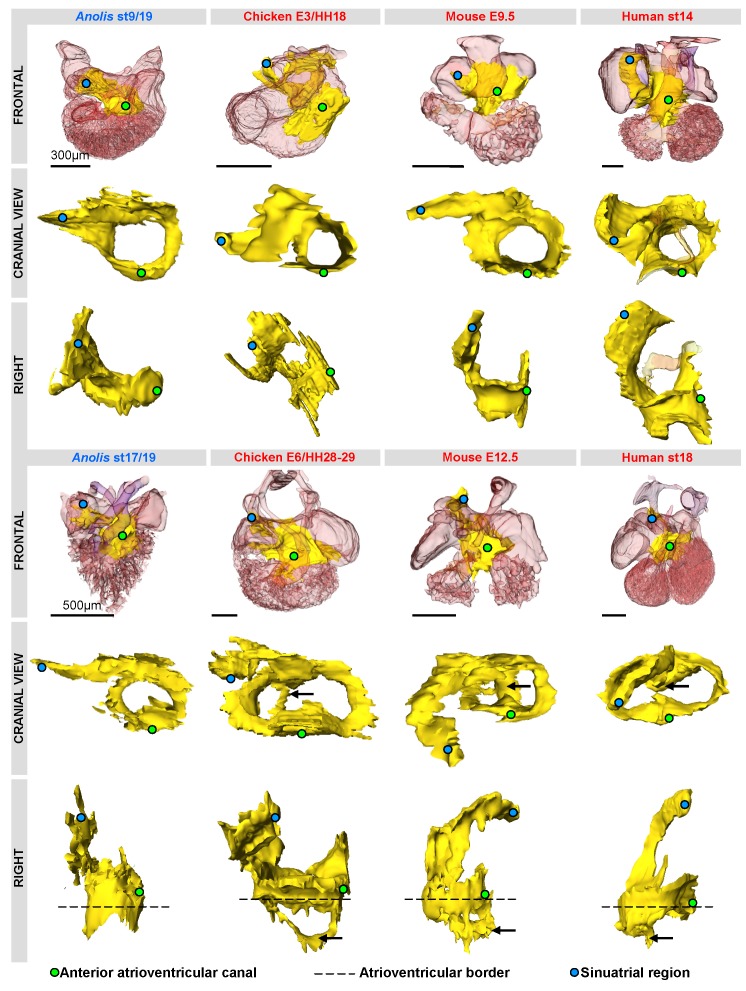
Three-dimensional reconstructions of *Tbx3* (yellow) expression in amniotes reveal a shared design. Reconstructions are based on in-situ hybridizations of serial sections, except in human (based on immunohistochemistry, modified from [57], [58]). The *Tbx3* domains are strikingly similar in the early phases of chamber formation (upper panel). The *Tbx3* expression of the *Anolis* ventricle is very similar to that associated with ventricular septation (black arrows) in the other amniotes (lower panel).

### Optical Mapping

Optical mapping was performed at 25°C in the ectothermic vertebrates and we used specific ringer solutions for zebrafish and *Xenopus* (in mmol/l: NaCl 115, Tris 5, NaH_2_PO_4_ 1, KCl 2.5, MgSO_4_ 1, CaCl_2_ 1.5, Glucose 5, pH adjusted to 7.2 with HCl) and *Anolis* (adopted from [21]; in mmol/l: NaCl 95, Tris 5, NaH_2_PO_4_ 1, KCl 2.5, MgSO_4_ 1, CaCl_2_ 1.5, Glucose 5, pH adjusted to 7.5 with HCl). For embryonic mouse hearts we used Tyrode’s solution at 37°C (in mmol/l: NaCl 128, KCl 4.7, CaCl_2_ 1.45, MgCl_2_ 0.6, NaHCO_3_ 27, NaH_2_PO_4_ 0.4, Glucose 11[pH maintained at 7.4 by equilibration with a mixture of 95% O_2_ and 5% CO_2_]). Excised hearts from sedated animals were incubated in the specific solution containing 15 mmol/l di-4-ANEPPS (voltage sensitive). Excitation light was provided by a 5-watt power LED (filtered 510±20 nm). Fluorescence (filtered >610 nm) was transmitted through a tandem lens system on CMOS sensor (100×100 elements; MICAM Ultima). Activation patterns were measured during sinus rhythm. Optical action potentials were analyzed with custom software.

**Figure 6:**
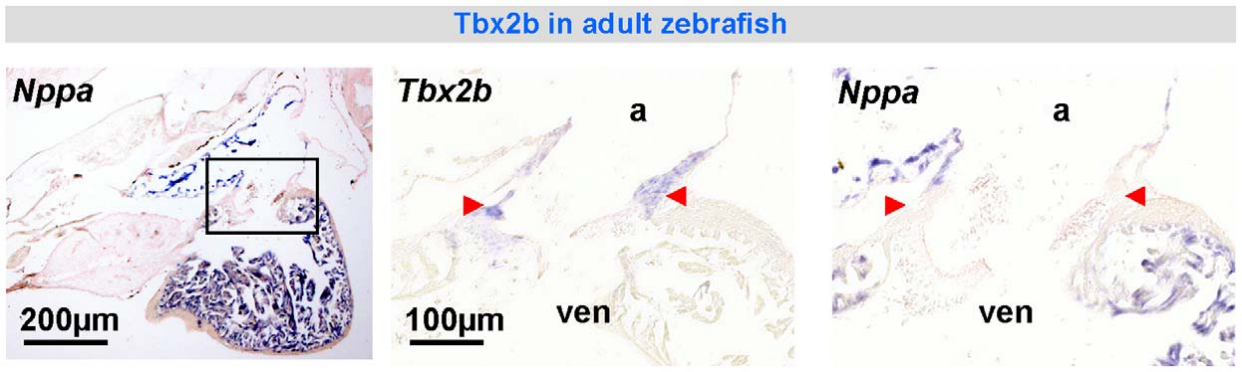
*Tbx2* expression in the atrioventricular canal of the formed heart of the zebrafish (*in-situ* hybridization). *Nppa* is expressed in a complementary pattern. a, atrium; ven, ventricle.

**Figure 7:**
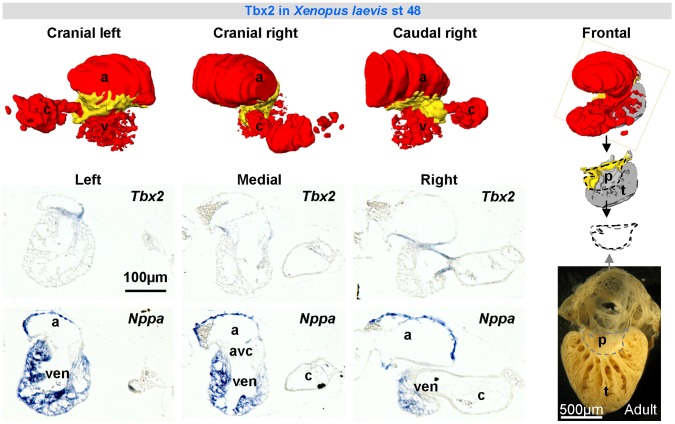
*Tbx2* expression (in-situ hybridization) in developing *Xenopus* (st 48). *Tbx2* is expressed in the atrioventricular canal and base of the myocardial outflow tract and complementary to *Nppa*, marker of chamber myocardium. In ectotherms primitive myocardium (p), remnants of the embryonic heart tube, can be recognized by its smooth surface as opposed to the trabeculated myocardium (t) formed during chamber formation. The primitive myocardium of the ventricular base of st 48 *Xenopus* hearts already has the adult configuration. The trabecular component is far from fully developed. a, atrium; avc, atrioventricular canal; c, conus arteriosus (myocardial outflow tract); p, primitive (atrabecular) myocardium; t, ventricular trabeculated myocardium; ven, ventricle.

### *In situ* Hybridization

All embryos and hearts were fixed in 4% paraformaldehyde for one day and then kept in 70% ethanol until imbedding in paraffin and then sectioned at 7–12 µm for *in situ* hybridization. Methodology of the non-radioactive in situ hybridization analysis has been described previously [22], [23] and so has probes for Zebrafish [16], Xenopus [24], [25], chicken [26]–[30] and mouse [26]. Probes for Anolis were made in house based on the following coordinates using UCSC Genome Browser on Lizard May 2010 (Broad AnoCar2.0/anoCar2) Assembly; *Tnnt2* (chr4∶131,362,830–131,375,015), *Bmp2* (chr1∶136,434,179–136,445,741), *Gja5* (chr3∶162,064,640–162,065,729), *Tbx3* (chrUn_GL343338∶1,255,354–1,255,812), *Tbx2* (regarded as *Tbx3*, chrLGb:3,261,791–3,267,757), *Tbx5* (chrUn_GL343338∶1,127,724–1,145,671). Briefly, we generated a cDNA library with standard TRIzol RNA extractions [31] from freeze-fixed specimens of developmental stages 5–9 and GOI cDNA were obtained by PCR amplification and cloned into pBluescript SK_ (Stratagene, La Jolla, CA). Digoxigenine-labeled antisense mRNA were then produced by in vitro transcription according to the manufacturer’s instructions (Roche, Mannheim, Germany).

**Figure 8:**
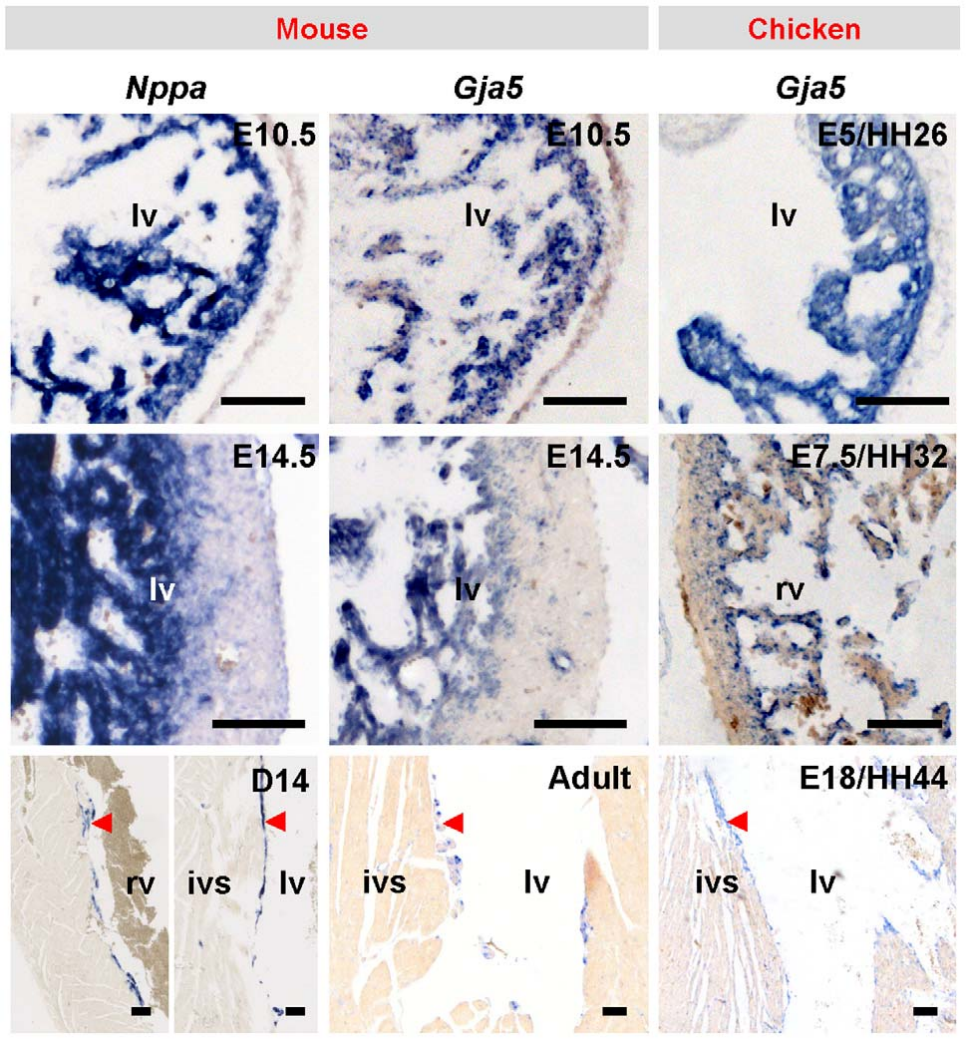
Development of compact walls. The development of the compact walls (*Nppa* and *Gja5* negative) of mammals and birds leaves the trabeculated myocardium (*Nppa* and *Gja5* positive) as a thin inner lining of the ventricular lumina in the fully formed hearts. *Nppa* is not expressed birds[36]. Scalebars, 100 µm. ivs, interventricular septum; lv, left ventricle.

**Figure 9:**
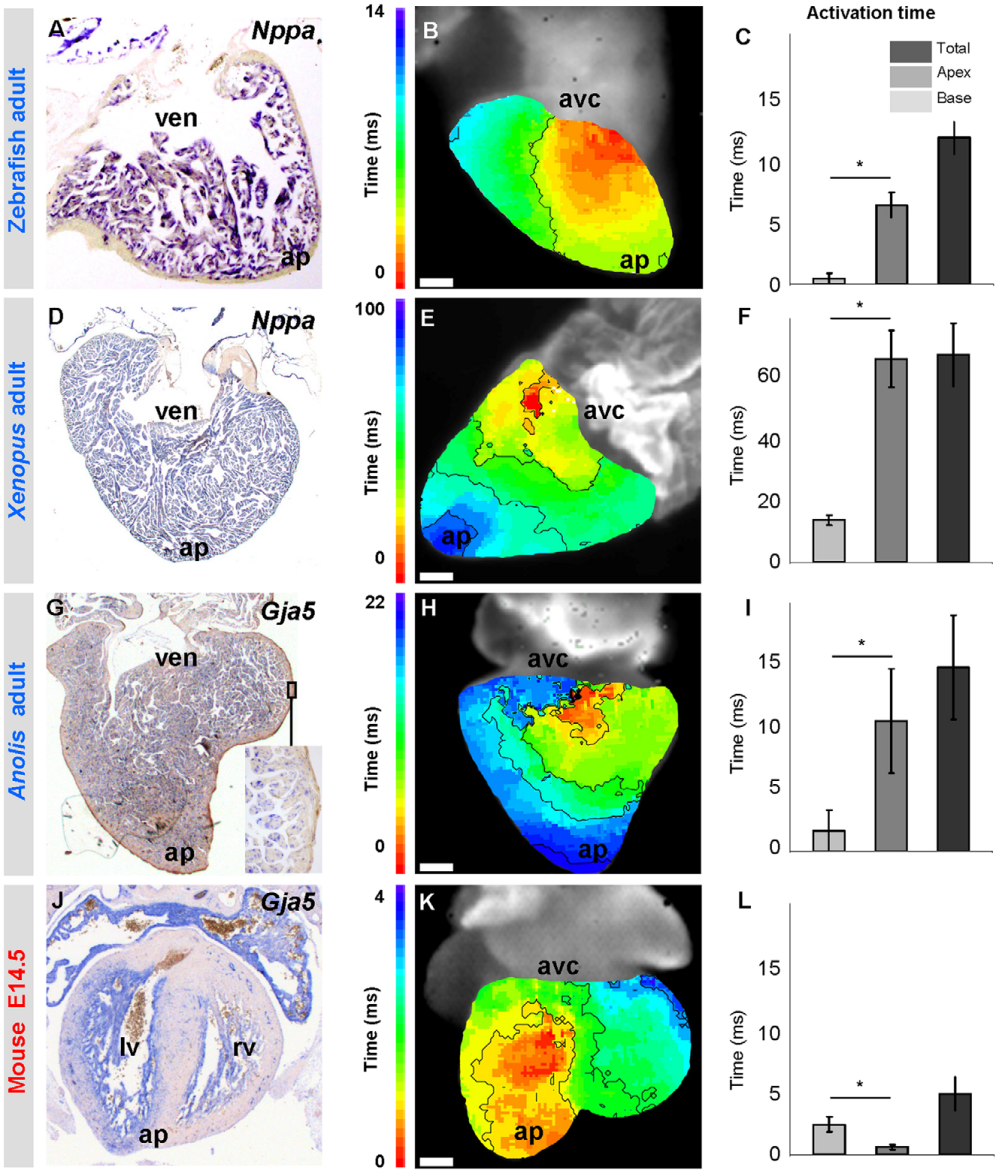
Trabeculated ventricles are activated from base to apex. (A, D, G, J) Markers of fast propagating myocardium (*Nppa* and *Gja5*) are homogenously expressed in the ventricular trabeculated myocardium from base to apex (ap). (B, E, H) Ventricular activation occurs from base to apex. Early activation is red, late activation is blue. Note that the time-colour coding in panel E is different from that in panels B and H. (K) In species with thick compact myocardium, surface breakthrough of the activation front is earlier in the apical region than in the base. (C, F, I, L) Graphs show the average activation time of the apex and base and the total ventricular activation time. Note that in zebrafish, Xenopus and Anolis, the ventricular base is activated earlier than the apex whereas in mice the ventricular base is activated later than the apex (* Significantly different (one-way ANOVA P<0.05)). n is 3, 6, 9 and 2, respectively. Scale bars in (B, E, H, K) indicate respectively 0.2, 1, 0.5 and 0.1 millimetre. avc, atrioventricular canal; ven, ventricle.

### 3D-reconstruction Protocol

7, 10 and 12 µm serial sections were stained by in situ hybridization and 3D reconstructions were performed as described previously using Amira® version 5.2 software [32]. The interactive 3D pdfs were created using Adobe Acrobat Pro Extended® version 9.3. The 3D pdf can be viewed with the freeware version: Adobe Reader® (version 9.3 or higher) with Javascript® enabled.

## Results and Discussion

In adult lizards, the sequential chamber contractions and an atrioventricular delay are well-established [33], but we found no insulating plane or insulated atrioventricular node in *Anolis* (Fig. 2). Instead, the atrioventricular canal was entirely myocardial (Fig. 3). This differs from the adult hearts of mammals and birds, where the atrioventricular myocardium has largely disappeared and an insulating plane of fibrous-fatty tissue has ingressed between the atria and ventricles except at the atrioventricular node and His bundle, which provide the sole electrical communication between the atria and the ventricles [12], [34], [35]. To explain the atrioventricular delay in reptiles, we hypothesized, therefore, that the atrioventricular canal of *Anolis* has a molecular phenotype that differs from that of the chambers.

**Table 1:** Ventricular activation pattern in ectothermic vertebrates investigated by optical mapping(^OP^), ECG(^ECG^), electrodes(^E^) or markers (^M^).

FISH	Base-to-apex	Left-to-right^1^	Apex-to-base
*Acipenser sturio*	Noseda et al, 1962^ E^		
*Ameiurus nebulosus*	Noseda et al, 1962^ E^		
*Anguilla anguilla*	Noseda et al, 1962^ E^, 1963^ ECG^		
*Cyprinus carpio*	Noseda et al, 1962^ E^		
*Danio rerio*	This study^ OP^		Chi et al., 2008^ OP^Sedmera et al 2003^ OP^
*Esox lucius*	Vaykshnorayt et al, 2011^ E^		
*Protopterus ethiopicus*	(Arbel et al 1977^ E, ECG^)^2^		(Arbel et al 1977^ E, ECG^) 2
*Salmo irideus*	Noseda et al, 1962^ E^		
*Scyliorhinus canicula*	Noseda et al, 1962^ E^		
*Scyliorhinus stellare*	Noseda et al, 1962^ E^		
**AMPHIBIA**			
*Bufo marinus*	Mullen 1974^ ECG^		
*Bufo typhonius*	Mullen 1974^ ECG^		
*Bufo vulgaris major*	Lewis 1916^ E^		
*Caecilia guntheri*	Peters and Mullen 1966		
*Eleutherodactylus buergeri*			Mullen 1974^ ECG^
*Pleurodeles waltii*			Noseda et la, 1963^ ECG^
*Rana catesbeiana* *	Dillon and Morad 1981^ OP^		Dillon and Morad 1981^ OP^
*Rana esculenta*	Vaykshnorayt et al 2008^ E^		
*Rana temporaria*	Azarov et al 2007^ E^Vaykshnorayt et al 2008^ E^, 2011^ E^		
*Telmatobius montanus*	Mullen 1974^ ECG^		
*Xenopus laevis*	Furman 1960^ ECG^This study^ OP^		Sedmera et al 2003^ OP^
**REPTILIA**			
Squamata (50 sp)	Mullen, 1967^ ECG^		
*Alligator mississippiensis*	Heaton-Jones and King 1994^ ECG^		Syme et al 2002 ^E^
*Alligator sinensis*	Zhao-Xian et al 1991^ ECG^		
*Anolis caroliniensis*	This study^ OP^		
*Boa constrictor*	Valentinuzzi et al 1969^ ECG^		
*Chrysemis sp*	Meek and Eyster, 1912^ E^		
*Crocodylus johnstoni* *	Christian and Grigg 1999 ^E^		Christian and Grigg 1999 ^E^
*Graptemys pseudogeographica*		Gray 1950^ M^	
*Pseudemys elegans*	Harris 1941^ E^		
*Pseudemys scripta*		Burggren 1978^ E^	
*Pseudemys troosti*		Gray 1950^ M^	
*Testudo graeca*	Lewis 1916^ E^	Burggren 1978^ E^	
*Sphenodon punctatus*	McDonald and Heath 1971^ ECG^		

In the formed hearts of mammals and birds, epicardial ventricular activation is from apex to base.

1 Left-to-right activation is only reported in broad-hearted turtles.

2 Arbel et al (1977) using ventrally placed electrodes find that as the apex becomes activated the current spreads towards the base. As they could not evaluate if the base was activated earlier, and since the zebrafish outflow region is indeed activated later than the apex their results may be in agreement with the present study.

* Species where first point of activation definitely was neither base nor apex. From [33], [59]–[81].

In embryos of mammals, birds, frog and fish, *Nppa* and/or *Gja5* mark the rapid propagating atrial and ventricular chamber myocardium, whereas the atrioventricular canal is negative for these markers [3], [15] (*Nppa* is lost in birds and all reptiles except turtles [36], [37]). Focusing on the atrioventricular gene programme during *Anolis* development, we found *Tbx3* and *Bmp2* to be expressed in the developing atrioventricular canal myocardium, exactly complementary to *Gja5* in the adjacent chambers (Fig. 4A–D). *Tbx5*, known to promote differentiation into *Nppa-* and *Gja5-*expressing chamber myocardium [38], was also present in the atrioventricular canal and the chambers (Fig. S2) [39].

**Figure 10:**
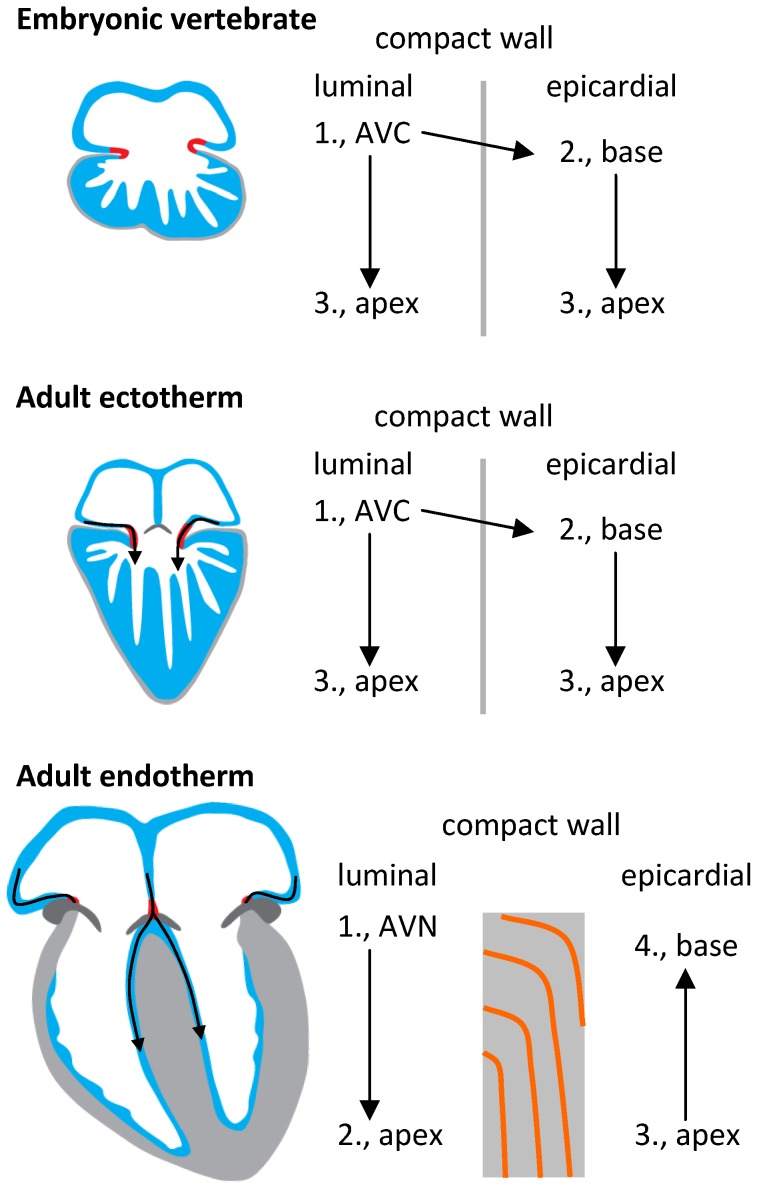
The trabecular myocardium is activated from base to apex in all vertebrates. (A–C) The trabecular myocardium gives rise to the His-Purkinje system in mammals and birds and remains activated from base to apex. (C) On the epicardial surface of septated and thick-walled ventricles, as in the formed hearts of mammals and birds, activation is seen to occur from apex to base and the luminal base-to-apex activation is obscured.

We then examined the adult atrioventricular region in *Anolis*, and observed that *Bmp2*, which in mammals and birds is expressed in the atrioventricular canal only at embryonic stages, remained expressed throughout ontogeny (Fig. 4B,F). *Tbx3*, which marks the cardiac conduction system in mature mammals [12], was found within the same *Bmp2*-positive atrioventricular domain (Fig. 4C,G). Three-dimensional reconstructions of the expression patterns of *Tbx3* in early developing hearts of *Anolis*, chicken and mammals revealed a striking resemblance (Fig. 5, Fig. S3). In all species, a similarly-shaped atrioventricular ring was observed. The *Tbx3* expression domain extended into the sinus venosus, marking the sinus node primordium. This expression pattern did not substantially change in lizards just prior to hatching, whereas in mammals and birds, it became more complex with further development as the morphology of the heart, and particularly the sinu-atrial region, changed (Fig. 5).

We then pursued this building plan of the atrioventricular canal to older vertebrate classes, represented by *Xenopus*, an amphibian and thus a non-amniotic member of tetrapods, as well as zebrafish. In both species, the atrioventricular canal is composed of myocardium in continuity with the atrium and ventricle (Fig. 3). An insulating plane and an insulated atrioventricular node were not found (Fig. 2). In adult zebrafish and stage 48 *Xenopus*, we found *Tbx2* in the atrioventricular canal and *Nppa* (whose spatio-temporal pattern strongly resembles that of *Gja5* in mammals) in the atria and ventricles, the patterns resembling those of *Tbx3* and *Gja5*, respectively, in the amniotic vertebrates (Figs. 6, 7). No cardiac *Tbx3* expression was found (Fig. S4).

Next, we examined the His-Purkinje system in *Anolis*. *Gja5* was used as marker for the mature His-Purkinje system conserved in mammals and chicken [10]. The developing His bundle does not express *Gja5* until late stages in mammals [40] and birds [41], but can nonetheless be identified very early by the expression of *Tbx3* [26]. In mouse [40] and chicken, the *Tbx3-*positive and *Gja5*-negative myocardium of the developing His bundle extends from the atrioventricular canal ventrally and dorsally into the ventricular myocardium and unto the crest of the ventricular septum (Fig. 5). The region formed by the dorsal and ventral extension and the crest is referred to as the primary ring [7]. The *Anolis* ventricle is not septated, but shows *Tbx3* expression into the ventricle ventrally and dorsally, indicating the presence of a primordial, but incomplete, primary ring that lacks the septal crest component (Fig. 5).

The ventricular wall in mammalian and avian embryos is composed of a trabecular inner layer and a thin compact outer layer. Initially, both of these layers express natriuretic peptides (*Nppa* and *Nppb*) and *Gja5* [27], [42], but halfway through development, *Nppa* and *Gja5* expression ceases in the strongly expanding compact layer. After birth (or hatching in birds) the expression of *Nppa* and *Gja5* is limited to the His-Purkinje network that eventually constitutes only a small fraction of the ventricular mass (Fig. 8) [10], [43]. The ventricular wall of fish, amphibian and reptilian hearts does not display such an overt distinction in expression pattern between an inner trabecular wall and a compact outer layer. Their ventricular wall typically is composed of a spongy or trabecular type of myocardium (Figs. 2, 3). In adult *Anolis* hearts, *Gja5* was homogenously expressed throughout the trabecular ventricular wall. This suggests an absence of tracts of preferential conduction leading to the ventricular apex and such condition resembles that of early mammalian and avian embryos (Fig. 9). Further back in evolution, as represented by *Xenopus* and zebrafish, we observed homogenous *Nppa* expression in their trabecular ventricular wall (Fig. 9A,D), while *Tbx3* did not identify a primordial atrioventricular bundle (Fig. S4).

Mammalian and avian hearts have an elaborate *Gja5*/*Nppa*-expressing ventricular conduction system that activates the ventricles from apex to base [42], [44], [45]. The homogenous *Gja5*/*Nppa* expression patterns in *Anolis*, *Xenopus* and zebrafish trabecular ventricles suggest that the electrical activation front may spread from the vicinity of the atrioventricular canal, *i.e.* the ventricular base, and reach the apex later. Such an activation pattern would be reminiscent of early embryonic mammalian and avian ventricles [45]. We used optical mapping to measure epicardial activation patterns in *Anolis*. The first point of activation always occurred in the cranial third of the ventricle, *i.e.* the base, and later at the apex (Fig. 9H). This activation pattern is consistent with most previous ECG and electrode investigations on reptiles and very similar to the activation patterns of chamber-forming hearts of mouse (E8–10) and chicken (E2–5) (Table 1) [45]–[47]. At these stages in mammals and birds, a morphologically distinct conduction system has yet to form and ventricular septation is only starting to take place [48], [49]. Assuming that the dorsal and ventral activation patterns share the same time point of activation at the apex, we could synchronize the activation patterns and infer that the dorsal base is activated prior to the ventral base in the ectothermic vertebrates (Fig. 9B,E). Dorsal activation of the ventricular base has been reported in chicken hearts prior to septation and seemingly occurs in embryonic mouse as well [42], [45]–[49]. In *Xenopus* and zebrafish, the activation front travels from the dorsal base to the apex. In *Anolis*, *Xenopus* and zebrafish the location of the first point of activation varied within the dorso-basal region (Fig. S5). The activation maps and expression data indicate that the trabeculated ventricular wall of the ectothermic vertebrates function essentially as an isotropic conduction network. Consistently, conduction on the luminal surface of the adult mammalian and avian ventricles through the *Gja5-*positive His-Purkinje system also proceeds from base-to-apex [49], [50]. On the epicardial side, however, the activation front reaches the apex first and then the base (Fig. 9J–L) [51]. In mammals and birds, the developmental change in activation pattern to apex-to-base observed at the epicardial side coincides with the development of the *Gja5*-negative compact ventricular wall, which therefore may contribute to this developmental change in activation pattern.

Hearts of ectothermic species and of embryos of endothermic species do not have anatomically marked conduction system components. In this study we used expression patterns of conserved genetic markers and identified molecular conduction system components in developing and adult lizards. We found them to be similar to the components in embryonic mammals and birds, indicating they constitute an integral part of the building plan of the heart. Therefore, the conduction systems found in mature mammals and birds most likely evolved from the components of this shared building plan, and did not evolved independently.

In mature birds and mammals, left-over traces of the atrioventricular canal muscle in addition to the atrioventricular node can be found. Birds have a well-developed right-sided atrioventricular ring bundle that communicates with the ventricle anteriorly through the so-called recurrent branch [4], [52]. The mammalian heart also maintains a molecularly distinct atrioventricular ring bundle above the insulating plane [12]. Interestingly, in congenital corrected transposition of the human heart, the insulating plane disrupts the normal posterior atrioventricular communication, whereas the anterior communication is abnormally maintained [53], [54]. The anterior part, then, resembles the recurrent branch of the bird heart.

The adult lizards and ectothermic vertebrates in general maintain important aspects of the embryonic vertebrate building plan. The *Bmp2/4-Tbx2/3-*positive, *Gja5/Nppa-*negative atrioventricular canal myocardium is maintained in adult ectothermic vertebrates. This provides an electrical insulation between atrium and ventricle in these hearts that lack an insulating plane of connective tissue. The developing hearts of mammals and birds have great tolerance to ischemia and regenerative potential, which is lost around birth [55], [56]. Interestingly, many ectothermic vertebrates (e.g. newts and zebrafish) retain the regenerative capacity and ischemia tolerance throughout life [6]. It is therefore tempting to speculate that the maintenance of important aspects of the embryonic programme in adult ectothermic vertebrates may be involved in the retention of these capacities.

Our study provides a plausible scenario of the evolution of the hearts of mammals and birds. The spongy myocardium of ectothermic adult vertebrates, as well embryonic mammals and birds allows for high ejection fractions and also serves to conduct the ventricular depolarization (Fig. 10). However, a transition to compact myocardium was necessary when pressure and heart rate increased. This rendered the early trabecules secondary on force generation, but available to differentiate into fibres of poor contractility and high propagation speeds. Furthermore, mammals and birds develop a compact ventricular septum whereby the early trabecules come to drape the septal surfaces and thus form the characteristic bundle branches of the His bundle (Fig. 10). Our study, therefore, suggests that the parallel evolution of virtually identical conduction systems and cardiac designs in birds and mammals can be traced back to the existence of a primordial conduction system of the ancestral reptile heart.

## Supporting Information

Figure S1 **Gene program of the developing atrioventricular canal in chicken**. *Tbx5*, known to induce *Gja5*, is present in the atrioventricular canal but *Gja5* is absent where *Bmp2* and *Tbx3* are expressed. la, left atrium; lv, left ventricle; ra, right atrium; rv, right ventricle.
(TIF)

Figure S2 **Gene program of the developing atrioventricular canal in** ***Anolis***. Despite expression of *Tbx5* the atrioventricular canal does not initiate chamber program and expresses the transcription repressor *Tbx3* along with *Bmp2*. avv, atrioventricular valves; la, left atrium; ra, right atrium; t, trachea (positive for *Tbx3*).
(TIF)

Figure S3 ***Tbx3*** **expression shown in interactive 3D pdfs**. Tbx3 expression (yellow) and lumen cast (red) of all models used in Figure 5.
(PDF)

Figure S4 ***Tbx3*** **expression (in-situ hybridization) in developing and adult** ***Xenopus***. *Tbx3* was only found outside the heart (*e.g.* developing trachea, t) of stage 40 embryos (top row). No *Tbx3* was only found in the heart of adults (lower row). a, atrium; avc, atrioventricular canal; c, conus; ven, ventricle; t, developing trachea (positive for *Tbx3*).
(TIF)

Figure S5 **Summary of individual ventricular activation maps**. Red marks the earliest epicardial breakthrough of the activation front which was consistently at the ventricular base. Earliest and latest activation (red and white dots respectively) from each specimen is projected onto one specimen.
(TIF)
