# Supplementary figures and images for: Identifying the Evolutionary Building Blocks of the Cardiac Conduction System
Source: PLoS One. 2012 Sep 11;7(9):e44231. doi: 10.1371/journal.pone.0044231 (PMC3439475; doi:10.1371/journal.pone.0044231)

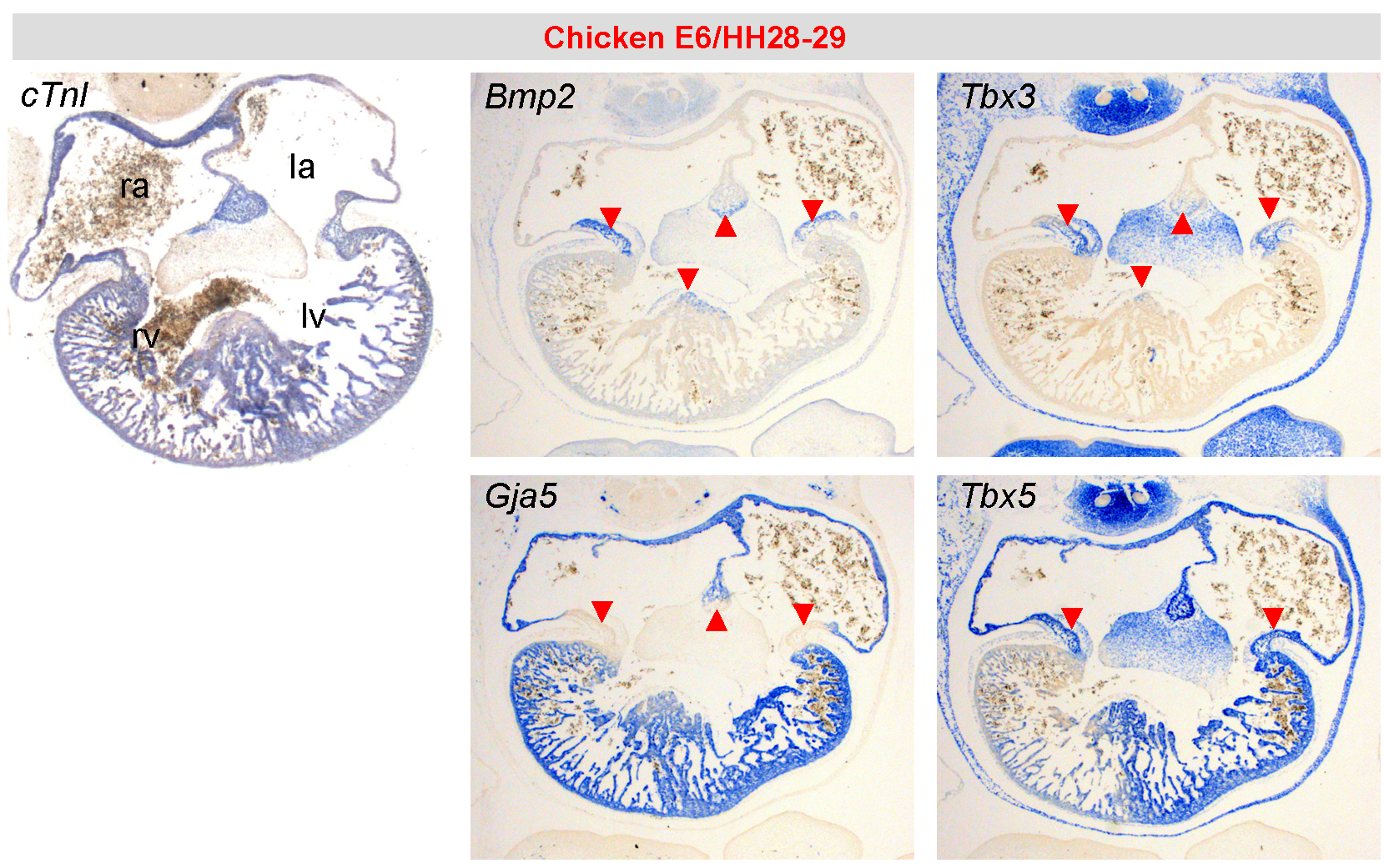

Supplement: Figure S1 — Gene program of the developing atrioventricular canal in chicken. Tbx5, known to induce Gja5, is present in the atrioventricular canal but Gja5 is absent where Bmp2 and Tbx3 are expressed. la, left atrium; lv, left ventricle; ra, right atrium; rv, right ventricle. (TIF) [file pone.0044231.s001.tif]

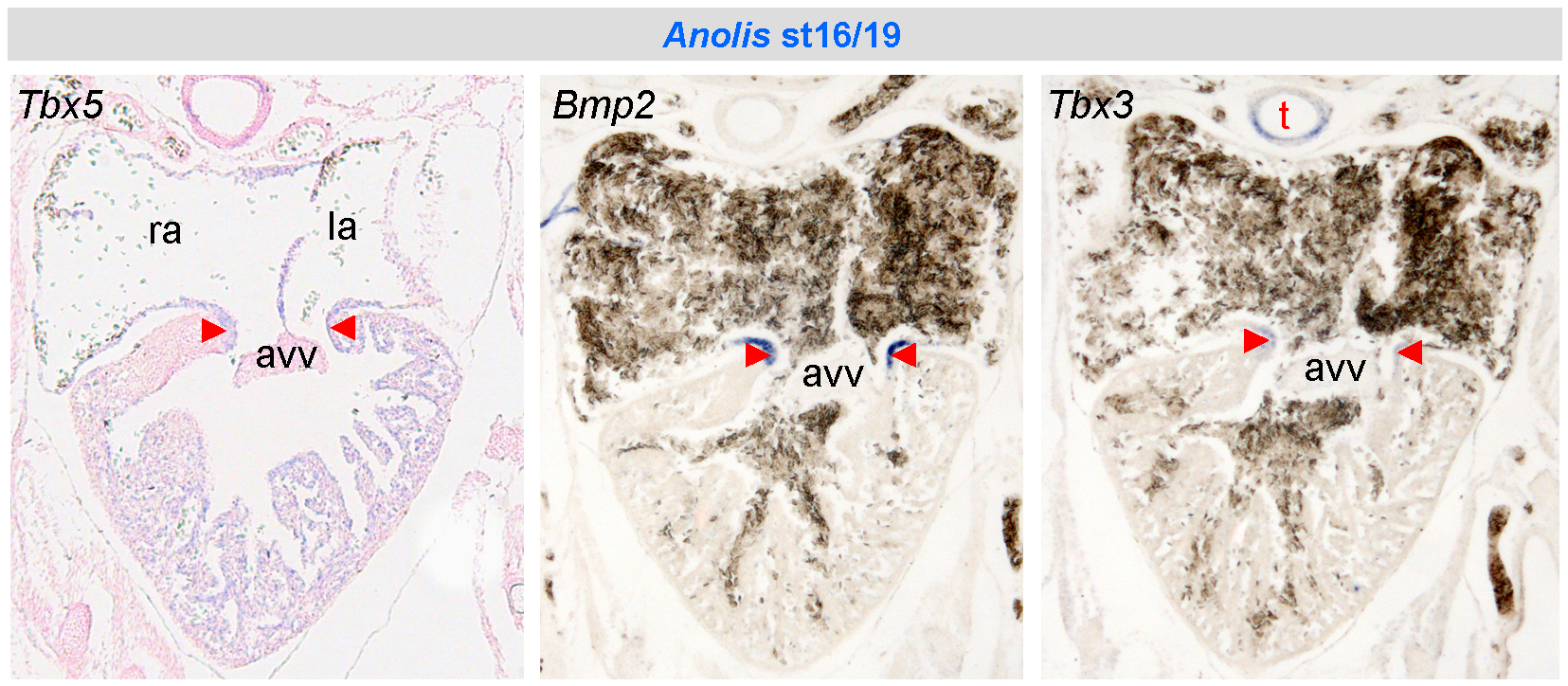

Supplement: Figure S2 — Gene program of the developing atrioventricular canal in Anolis. Despite expression of Tbx5 the atrioventricular canal does not initiate chamber program and expresses the transcription repressor Tbx3 along with Bmp2. avv, atrioventricular valves; la, left atrium; ra, right atrium; t, trachea (positive for Tbx3). (TIF) [file pone.0044231.s002.tif]

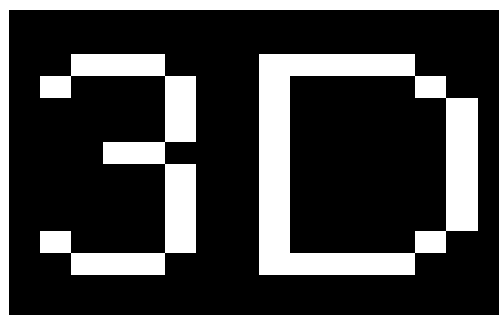

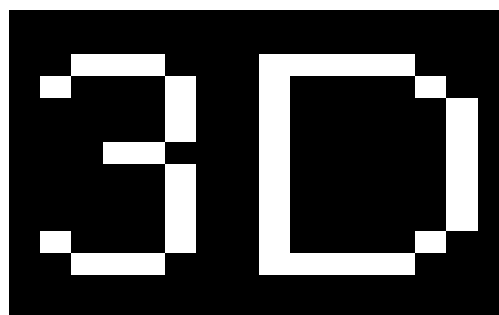

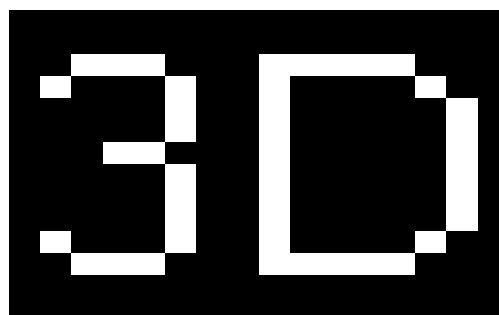

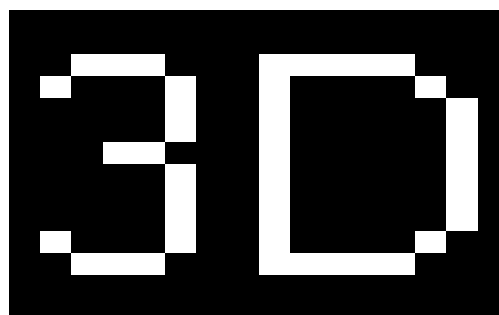

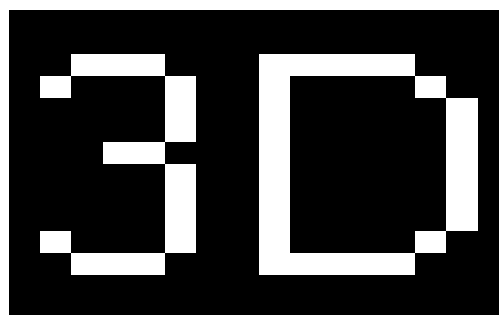

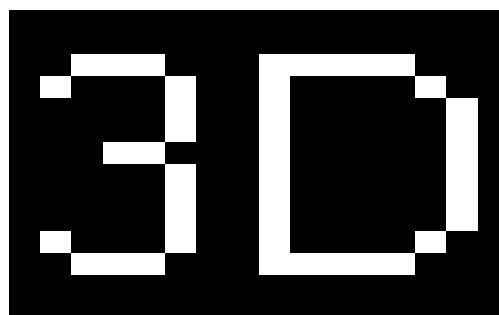

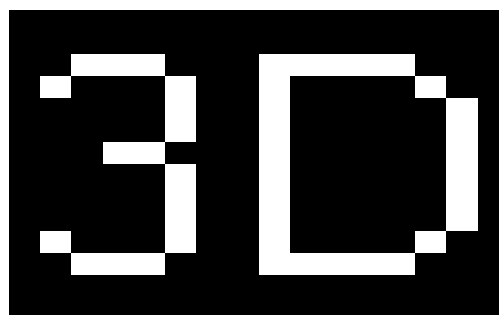

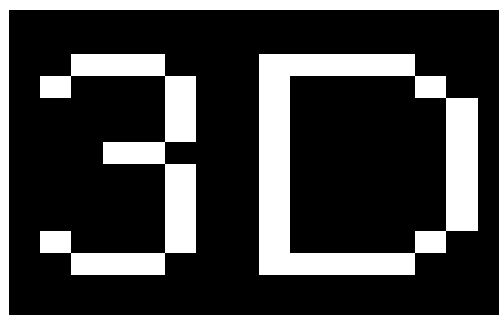

Supplement: Figure S3 — Tbx3 expression shown in interactive 3D pdfs. Tbx3 expression (yellow) and lumen cast (red) of all models used in Figure 5. (PDF) [file pone.0044231.s003.pdf]

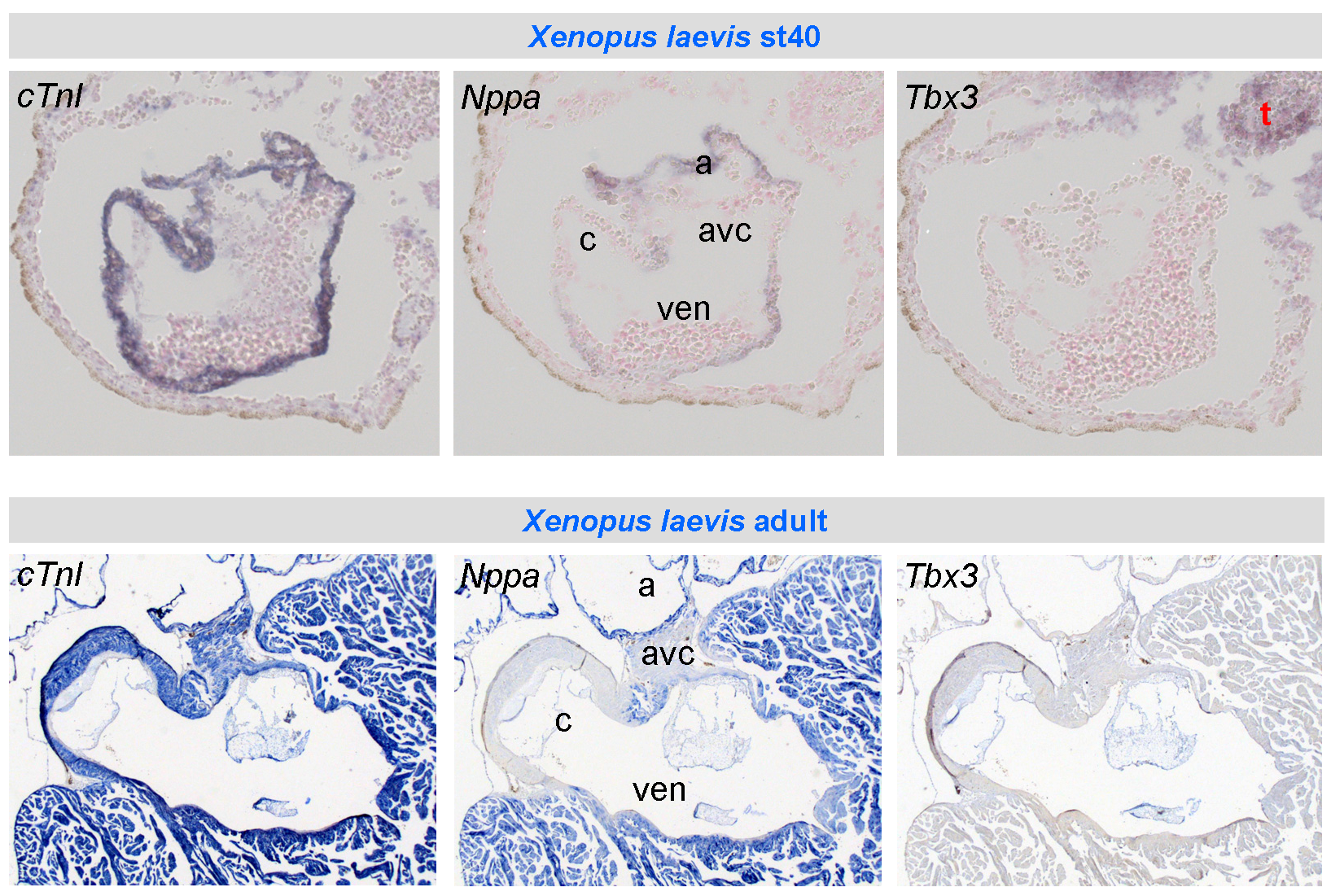

Supplement: Figure S4 — Tbx3 expression (in-situ hybridization) in developing and adult Xenopus. Tbx3 was only found outside the heart (e.g. developing trachea, t) of stage 40 embryos (top row). No Tbx3 was only found in the heart of adults (lower row). a, atrium; avc, atrioventricular canal; c, conus; ven, ventricle; t, developing trachea (positive for Tbx3). (TIF) [file pone.0044231.s004.tif]

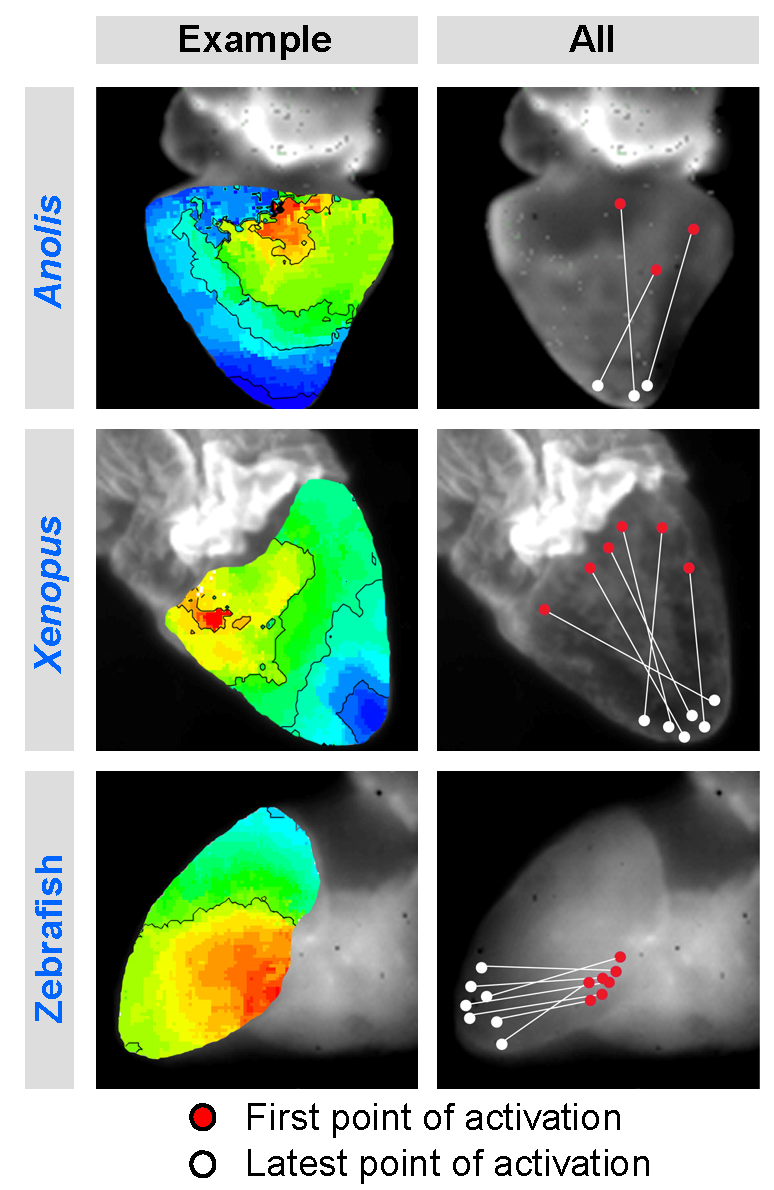

Supplement: Figure S5 — Summary of individual ventricular activation maps. Red marks the earliest epicardial breakthrough of the activation front which was consistently at the ventricular base. Earliest and latest activation (red and white dots respectively) from each specimen is projected onto one specimen. (TIF) [file pone.0044231.s005.tif]
